# Plants with potential use on obesity and its complications

**DOI:** 10.17179/excli2015-186

**Published:** 2015-07-09

**Authors:** Claudia I. Gamboa-Gómez, Nuria E. Rocha-Guzmán, J. Alberto Gallegos-Infante, Martha R. Moreno-Jiménez, Blanca D. Vázquez-Cabral, Rubén F. González-Laredo

**Affiliations:** 1 Instituto Tecnológico de Durango, Felipe Pescador 1830 Ote., 34080 Durango, Dgo., México

**Keywords:** obesity, obesity complications, anti-obesity plants, phytochemicals, alternative treatments

## Abstract

Obesity is the most prevalent nutritional disease and a growing public health problem worldwide. This disease is a causal component of the metabolic syndrome related with abnormalities, including hyperglycemia, dyslipidemia, hypertension, inflammation, among others. There are anti-obesity drugs, affecting the fundamental processes of the weight regulation; however they have shown serious side effects, which outweigh their beneficial effects. Most recent studies on the treatment of obesity and its complications have focused on the potential role of different plants preparation that can exert a positive effect on the mechanisms involved in this pathology. For instance, anti-obesity effects of green tea and its isolated active principles have been reported in both *in vitro* (cell cultures) and *in vivo* (animal models) that possess healthy effects, decreasing adipose tissue through reduction of adipocytes differentiation and proliferation. A positive effect in lipid profile, and lipid and carbohydrates metabolisms were demonstrated as well. In addition, anti-inflammatory and antioxidant activities were studied. However, the consumption of green tea and its products is not that common in Western countries, where other plants with similar bioactivity predominate; nevertheless, the effect extension has not been analyzed in depth, despite of their potential as alternative treatment for obesity. In this review the anti-obesity potential and reported mechanisms of action of diverse plants such as: *Camellia sinensis, Hibiscus sabdariffa, Hypericum perforatum, Persea americana, Phaseolus vulgaris, Capsicum annuum, Rosmarinus officinalis, Ilex paraguariensis, Citrus paradisi, Citrus limon, Punica granatum, Aloe vera, Taraxacum officinale *and* Arachis hypogaea* is summarized. We consider the potential of these plants as natural alternative treatments of some metabolic alterations associated with obesity.

## Introduction

Obesity is now the most prevalent nutritional disease and a growing public health problem worldwide. The disease has acquired epidemic proportions projected to reach 2.3 billion of overweight adults and 700 million obese adults, respectively by 2015 (Malik et al., 2013[88]).

Overweight is an established risk factor for type 2 diabetes and cardiovascular diseases, where the central and causal component is the metabolic syndrome (Montague and O’Rahilly, 2000[96]). This is a series of metabolic abnormalities including hyperglycemia, dyslipidemia, hypertension, inflammation, oxidative stress, among others (Grundy et al., 2004[55]). 

Perturbed intravascular lipid and lipoprotein metabolism is a common feature of obesity, leading to a dyslipidemia involving elevated plasma concentrations of triglycerides and apolipoprotein (apo) B-containing lipoproteins (very low density lipoprotein: VLDL and low density lipoprotein: LDL) and subnormal levels of high density lipoprotein: HDL), a lipid phenotype associated with accelerated atherosclerosis and high cardiovascular risk (Ståhlman et al., 2013[133]). 

The metabolic syndrome consequence of obesity is associated in part by the alterations in adipose tissue; however, it is not the only one involved, participating other tissues such as liver, among others (Hirosumi et al., 2002[63]). Adipocytes produce a variety of biologically active molecules (Saltiel and Kahn, 2001[121]) collectively known as adipocytokines or adipokines (e.g. Tumor necrosis factor alpha: TNFα and interleukin-6: IL6). In obesity, hypertrophy of adipocytes could lead to regions of hypoxia, which could instigate an inflammatory response (Ye et al., 2007[152]). This phenomenon could alter the adipokine profile via reduced adiponectin expression (Wree et al., 2012[149]) and increased leptin (Skurk et al., 2007[128]). Increased TNF-α and IL-6 production by adipose tissue infiltrated-macrophages were associated with atherogenic effects and insulin resistance (Mracek et al., 2010[99]; Skurk et al., 2007[128]). 

Insulin resistance is one of the most common alterations on obesity (Boden, 2011[18]), and is closely linked with diseases such as type 2 diabetes, fatty liver, atherosclerosis, hypertension, among others (Bray, 2004[21]). The alterations on insulin metabolism involve glucose transport, glycogen synthesis, and changes on lypolisis (Boden, 2011[18]). 

There are different pharmacological treatments; for example, Orlistat (Xenical), which reduces intestinal fat absorption through inhibition of pancreatic lipase (Thurairajah et al., 2005[135]), and Sibutramine (Reductil) that is an appetite suppressant (Tziomalos et al., 2009[139]). Furthermore, the consumption of anti-obesity drugs provokes collateral effects such as alterations in blood pressure, headaches, among others (de Simone and D'Addeo, 2008[33]; Karamadoukis et al., 2009[73]; Slovacek et al., 2008[129]; Thurairajah et al., 2005[135]). 

Most recent studies on the treatment of obesity have focused on the potential role of plants used for obesity and its metabolic disorders treatments, exerting a positive effect on lipid and glucose metabolism, and anti-inflammatory activity. For instance, anti-obesity effects of green tea; its bioactive compounds have demonstrated in both *in vitro* (cell cultures) and *in vivo* (animal models) that possess a healthy effect, decreasing adipose tissue through reduction of adipocytes differentiation and proliferation. A positive effect in lipid profile, and lipid and carbohydrates metabolisms, and anti-inflammatory activities were observed as well (Tipoe et al., 2007[137]; Wolfram et al., 2006[148]). However, the consumption of green tea in Western countries is not that common as other plants with similar effects. For this reason and due to the huge worldwide public health problem that obesity represents, we have summarized the information on some traditional plants with anti-obesity potential based in the current scientific literature.

## Epidemiology of Obesity

Obesity is a clinical condition, whose prevalence has sharply increased in the last decades in Western countries and, later on, worldwide. Obesity has been defined as a pandemic and one of the major health problems ever.

The prevalence of overweight in children is greater than 25 %, whereas in adults, it is greater than 50 % (Barquera et al., 2013[12]). In Mexico the incidence of obesity has been increased in the past 30 years, where the female population has been the most affected group (approximate 1.5 % points); while, the preschool children have shown lower morbidity (0.3 % points) (Barquera et al., 2013[11]). This prevalence is associated with the extreme dietary changes occurred in Western countries such as Mexico, which may be summarized as an increase in consumption of non-nutritional carbohydrates, especially sugar-sweetened beverages that has led to an increasing rate of chronic diseases related to obesity such as diabetes and cardiovascular diseases.

### Pathophysiology of obesity: adipose tissue

Human adipose tissue is divided into brown and white adipose tissues. The main activity of brown adipocytes is the regulation of thermogenesis. This is due to brown adipocytes cells that have numerous mitochondria; therefore, they express high concentration of uncoupling protein 1 (UCP-1) (Dulloo et al., 2010[38]). On the other hand, adipocytes are responsible for fat storage. The structure of adipose tissue includes macrophages, fibroblasts, pre and mature adipocytes (Sánchez et al., 2005[122]). 

High caloric diet promotes hyperplasia and hypertrophy of adipocytes. When a hypertrophy occurs, the size of adipocytes increases and the diffusion of oxygen is affected leading to hypoxia (Poulain-Godefroy et al., 2008[112]). As a result of hypoxia, adipocytes express the factor hypoxia-inducible (HIF-1a) and the unfolded protein response in the endoplasmic reticulum (Goossens, 2008[51]). HIF-1a modulates the genes involved with the expression of pro-inflammatory cytokines, for example: leptin, vascular endothelial growth factor (VEGF), among others (Goossens, 2008[51]). Other feature of hypertrophic adipocytes is that they show poor sensibility to insulin, due to the reported affectation of membrane receptors as consequence of obesity. This contributes to inflammation through diapedisis of monocytes to visceral stroma (Deng and Scherer, 2010[34]). 

The lower insulin sensitivity in hypertrophic adipocytes, is related in part to the non-esterified fatty acids, glycerol, hormones, and other factors released by these cells. The association of alterations in pancreatic islet β-cells and insulin resistance in insulin-dependent tissues lead to type 2 diabetes (Kahn et al., 2006[70]).

### Obesity and its complications: inflammation, diabetes type 2, and cardiovascular disease

There are multiple molecular mechanisms linking obesity to its complications such as type 2 diabetes mellitus, hypertension, hypercholesterolemia, atherosclerosis, non-alcoholic fatty liver disease, among others. However, inflammation is a common feature that has been implicated in the pathophysiology of many obesity-associated disorders. For instance, the main biological effect of leptin is the control of adipose tissue growth via its central nervous system action (Berglund et al., 2012[14]). In obesity there are increased levels of leptin, resulting in an apparent decrease of its anorexigenic effects and weight loss, result of a mechanism of resistance to it (Fonseca-Alaniz et al., 2007[44]). Leptin acts directly on macrophages increasing phagocytic activity and pro-inflammatory cytokine production. Leptin also stimulates proliferation and migration of endothelial cells and smooth muscle cells, thus favoring the development of atherosclerosis (Cachofeiro et al., 2006[24]).

Another example of the relation of disorders of obesity and inflammation is interleukin 6 (IL-6). This molecule is associated with the regulation of insulin signaling, decreasing the expression of the insulin receptor substrate 1 (IRS_1_), and upregulating the expression of suppressor of cytokine signaling (SOCS-3) (Rieusset et al., 2004[119]). SOCS-3 belongs to a family of inflammatory mediators that contributes to obesity-induced insulin resistance, which constitutes a negative feedback pathway in cytokine signaling (Lubis et al., 2008[86]).

The definition of insulin resistance implies a lower sensitivity to insulin by cells and alterations on glucose metabolism (for example: uptake or storage). In obesity and type 2 diabetes a decrease in insulin-stimulated glucose transport is observed; in addition, alterations on metabolism of adipocytes, skeletal muscle, and hepatic glucose output are also reported (Reaven, 1995[117]). Even though, insulin resistance is closely associated with several chronic diseases such as atherosclerosis, hyperlipidemia, and hypertension, among others (Reaven, 1995[117]; Meigs et al., 2007[91]).

The metabolic insulin resistance is usually accompanied by compensatory hyperinsulinemia, which may stimulate unaffected MAPK-dependent pathways in the vasculature, leading to decreased production of oxide nitric (NO) and increased secretion of endoteline 1 (ET-1) (Eringa et al., 2004[41]; Mather et al., 2004[90]). As a consequence, vasoconstriction of resistance arteries and terminal arterioles occurs, causing impaired regulation of muscle perfusion, glucose uptake, and blood pressure (Levy et al., 2001[82]), resulting in cardiovascular disorders.

### Plants used as an alternative for obesity and its complications

The complex pathogenesis of obesity indicates the need of different intervention strategies to confront this problem. Herbal supplements and diet-based therapies for weight loss are among the most common complementary and alternative medicine modalities (Barnes et al., 2002[10]). As an alternative treatment of obesity and its complications, in the market are a variety of natural products that includes medicinal plants, either as pure compounds or as extracts (Hasani-Ranjbar et al., 2013[57]).

Different plants contain a large variety of several components with different anti-obesity effects on body metabolism and fat oxidation, and for this reason have been investigated and reported to be useful in treatment of obesity, diabetes and other chronic diseases (Hasani-Ranjbar et al., 2009[58], 2010[59]).

### Camellia sinensis

The most studied plant due to its wide range of effects including anti-obesity and antioxidant properties is *Camellia sinensis*. Various studies have shown its beneficial effects on obesity. For example, Tian et al. (2013[136]) reported that a treatment with tea phytochemicals (0.8, 1.6, 3.2 g/L) administered as drinking water to male Wistar rats fed with high-fat diet for 26 weeks attenuated visceral adipose tissue accumulation (approx. 40 % at all doses). Similarly, green tea extract (400 mg/kg body weight/day) administered concomitantly in obese mice induced with high-fat diet for 8 weeks, increased the hormone-sensitive lipase and perilipin in mesenteric adipose tissue (Cunha et al., 2013[30]). This was associated to reduced body weight (16 %) and adipose tissue gain (64 % on epididymal adipose tissue) compared with the obese control. 

A recent study showed that not only the leaf of *C. sinensis* has anti-obesity effect, but also the fruit peel, which is considered an agricultural waste (Chaudhary et al., 2014[28]). The administration of ethanolic green tea fruit peel extract (100 mg/kg/d) on high-fat diet feed female Sprague-Dawley rats for 50 days significantly decreased the body weight (approx. 25 %) and white adipose tissue fat-pad weights (74 %) than the obese control. 

Different anti-obesity acting mechanisms have been reported for *C. sinensis*, including inhibition of pancreatic lipase (Grove et al., 2012[54]; Yuda et al., 2012[155]), appetite-repression activity (Moon et al., 2007[97]; Wolfram et al., 2006[148]), adipogenesis down-regulation (Lu et al., 2012[85]), thermogenesis (Hursel et al., 2011[65]), lipid metabolism (Axling et al., 2012[6]; Lu et al., 2012[85]), among others.

The chemical compounds with the presumed anti-obesity effect are especially catechin type polyphenols. An *in vivo* animal model study reports that Epigallocatechin 3-gallate, which is the major catechin like flavonoid in *Camellia sinensis* reduces body weight gain (Bose et al., 2008[19]). In human experiments, acute ingestion of green tea extracts increases the proportion of whole-body fat utilization by augmenting oxidation and lipolysis (Basu et al., 2010[13]).

An increasing number of studies have shown beneficial effects of green tea in metabolic disorders associated to obesity such as inflammation, metabolic syndrome, type-2 diabetes and cardiovascular disease. For example, Park et al. (2012[109]) reported that green tea extract suppresses nuclear factor kappa-light-chain-enhancer of activated β cells (NFkβ) activation and inflammatory responses in diet-induced obese rats with nonalcoholic steatohepatitis. These anti-inflammatory activities of green tea extract were also accompanied by improvements in tissue glutathione status, mitigated the development of non-alcoholic steatohepatitis in an NFkB-dependent manner by improving tissue redox status. 

On the other hand, Park et al. (2011[108]) reported that *Camellia sinensis* attenuates fat liver through decreased lipogenesis and improved hepatic oxidative stress conditions in ob/ob mice. Also, treatment with green tea polyphenols administered to male fat Wistar rats decreased total cholesterol, LDL, and triglycerides serum concentrations than obese controls at any experimented doses (Tian et al., 2013[136]).

Due to all this evidence, research in depth of the different health properties of *C. sinensis *continues. Additionally, in order to understand the effects and mechanisms of action of others plant material with similar effects is common observed to *C. sinensis* as reference of anti-obesity alternative. 

### Hibiscus sabdariffa

This plant is the second most studied after green tea and commonly used as a local soft drink against inflammation, hypertension, and liver disorders. Previous reports showed that aqueous extract of *Hibiscus sabdariffa* calyces (120 mg/kg/day) for 60 days decreases body weight gain (22 %) and liver steatosis in monosodium glutamate induced obese mice (Alarcon-Aguilar et al., 2007[2]). Similarly, Gamboa-Gómez et al. (2014[47]) reported that infusions (1 % w/w) of *H. sabadariffa* (*ad lib.*) for 16 weeks in diet-induced obese rats decreased body (10 %) and adipose tissue weights (29 %) compared with the obese controls. 

In clinical trials, an anti-obesity effect of *H. sabadriffa* was also reported. The consumption for 12 weeks of *H. sabdariffa *extract reduced body weight, BMI, body fat and the waist-to-hip ratio in subjects with a BMI ≧ 27 and aged 18-65 (Chang et al., 2014[27]).

Several anti-obesity acting mechanisms were reported for *H. sabdariffa* included the down-regulation of genes involved in lipid metabolism such as free fatty acid synthase (FAS) and Sterol Regulatory Element-Binding Proteins (SREBP1c) (Gamboa-Gómez et al., 2014[47]). Additionally, Kim et al. (2003[77]) reported that consumption of *H. sabdariffa* attenuates the hypertrophy of adipocytes through the inhibition of lipid droplet accumulation; this is in association to the inhibition of adipocyte differentiation. Also, a down-regulation of pancreatic lipases and fatty acid synthases have been reported (Kim et al., 2003[77]). 

The main phytochemicals with biological effect reported in *H. sabdariffa *are phenolic and flavonoids compounds with estimated contents of 58.80 and 13.57 mg/g dried flower, respectively; whereas, delphinidin-3-sambubioside, cyanidin-3-sambubioside, kaempferol-3-glucoside, protocatechuic acid, and chlorogenic acid are included in its chemical composition (Peng et al., 2011[110]).

In addition to its anti-obesity activity, several studies have reported beneficial effect on complications associated to obesity. Low and high doses (100 and 200 mg/kg) of methanolic *H. sabdariffa *extracts in type 2 diabetic rat model for 2 weeks improved blood glucose (52 and 57 % less, respectively), ameliorating insulin resistance (Peng et al., 2011[110]). It has also reduced serum triglycerides (approx. 12 %), total cholesterol (10 and 15 %, respectively), and the risk ratio of LDL/HDL (approx. 15 and 38 % less, respectively). In addition to its antioxidative effect, *H. sabdariffa* suppressed the formation of advanced glycation end product receptor (approx. 38 % less) and connective tissue growth factor which could be pathogenic biomarkers in type 2 diabetes-associated vasculopathy (Peng et al., 2011[110]).

On the other hand, Ali et al. (2014[3]) reported that treatment with *H. sabdariffa* extracts (rats dose: 125 or 60 mg/kg; human dose: 125 mg/kg/day) on spontaneously hypertensive rats (for one week) and patients diagnosed with metabolic syndrome (for four weeks) significantly reduced blood pressure after the intervention period in the form of 24-h systolic blood pressure. In addition, *H. sabdariffa* extracts significantly reduced the amount of TNF-α-induced NFkβ and the reactive oxygen species production. Also, they observed that *H. sabdariffa* extract (40 g/mL) in humans, up-regulate the expression of oxide nitric synthase (eNOS), and therefore a higher concentration of oxide nitric was observed.

This body of evidence interpreted together, suggests that *H. sabdariffa* has great potential to reduce not only obesity, but also diseases associated with cardiovascular disease. For this reason, further studies are suggested.

### Hypericum perforatum

St John's wort (*Hypericum perforatum* L.) is an herbaceous perennial plant, native to Europe and Asia, and later introduced into America, where it has been naturalized. *H. perforatum *is traditionally used for its health benefits associated to its sedative potential in alterations such as depression, anxiety, and neuralgia, among others (Newall et al., 1996[104]). Several studies report that *H. perforatum *has anti-obesity activity. For example, at 100 and 200 mg/kg of *H*. *perforatum* orally administered as suspension in 0.3 % carboxymethyl cellulose for 15 days in high-fat-fed rats reduced body weight (approx. 12 and 15 %, respectively) compared with controls (Husain et al., 2011[66]). Similarly, Hernández-Saavedra et al. (2013[60]) demonstrated that body weight gain was prevented (8 %) in obese rats fed with fructose and high saturated fat diet by *H*.* perforatum *infusion (*ad lib.*) treatment for 12 weeks; in addition, a significant reduction of adipocyte volume (62 %), and fat content (28 %) than obese controls was observed.

One anti-obesity acting mechanism reported for *H. perforatum *is the quantity of serotonin present within synaptosomes and inhibiting the synaptosomal uptake of serotonin (Husain et al., 2011[66]; Muller, 1997[100]). This increased level of serotonin reduces the food intake and suppresses the appetite (Yegorova and Jiang, 2002[153]).

Among the compounds with biological activity reported for *H. perforatum *are included hyperforin, hypeicin, flavonoids (kaempferol, quercetin, luteolin, isoquercitrin, quercitrin, rutin, among others), and condensed tannins (proanthocyanidins) are also present (Nahrstedt and Butterweck, 1997[101]). 

Moreover, *H. perforatum *has shown activity against complications associated to obesity. Husain et al. (2011[66]) reported that *H. perforatum *hydroalcoholic extract (50 %) significantly inhibited the increase in plasma glucose (85 %) and insulin (89 %) caused by fructose feeding, and 200 mg/kg of this extract significantly decreased plasma glucose (29 %) and insulin (12 %) level compared to fructose-fed control group. In addition to these results, *H. perforatum* dose dependently and significantly decreased total cholesterol (16 %) and triglycerides levels (18 %), while HDL (14 %) was increased compared to vehicle-treated fructose fed rats.

Similarly, Hernández-Saavedra et al. (2013[60]) reported that treatment of *H. perforatum* infusion ameliorated hyperglycemia in obese rats maintaining glucose levels under 100 mg/dL and insulin values similarly to the healthy control. Additionally, *H. perforatum *treatment group had the lowest leptin (21 %) and TNF-α (43 %) values as well as a substantial increase in the adiponectin (67 %) concentration than the obese control.

All this evidence is clearly relevant for assessing anti-obesity potential of *H. perforatum*, but it does not lend it all to establishing effect in human. Therefore, clinical investigations are required.

### Persea americana

Avocado (*Persea americana*) belongs to the laurel family Lauraceae and is native from Central and South America. Its cultivation has spread throughout Africa, Asia, Europe and United States. It has been reported that avocado leaves are traditionally used as an alternative treatment for diseases such as hypertension, diarrhea, pyorrhea, hemorrhage, sore throat; whereas the fruit is consumed as food (Brai et al., 2007[20]).

Anti-obesity effects have been reported for both the leaf and fruit. Brai et al. (2007[20]) reported that treatment with aqueous and methanolic extracts of *P. americana* leaves (10 mg/Kg) for 8 weeks in hypercholesterolemic albino rats (feed with diet containing 20 % groundnut oil, 0.5 % cholesterol, and 0.25 % cholic acid) caused 25 % reduction in the body weight gain compared to the control.

Additionally, Naveh et al. (2002[103]) studied defatted avocado pulp (100 g/Kg diet) in rats fed with cholesterol for 28 days. It was reported that body weight gains decreased (26 %) in the avocado-fed groups compared with a cellulose control group; they concluded that the defatted avocado pulp affected food consumption. Other anti-obesity mechanism was studied by Padmanabhan and Arumugam, (2014[106]). They reported that hydro-alcoholic fruit extract of *P. americana *(100 mg/kg body weight) for 14 weeks, significantly reduced the weight gain (24.77 %) and body mass index (17.92 %) in rats fed at high-fat diet (23 % fat). This effect was attributed to the up-regulation of Peroxisome proliferator-activated receptor gamma (PPAR-γ). 

Few information of alternative mechanism of action is available and this is an important area requiring additional data. Clinical intervention trials should be conducted to verify the mechanisms of action of *P. americana* observed in animal studies.

Major chemical constituents of the plant parts from *P. americana* have been well summarized (Ding et al., 2007[36]). These compounds may be divided into alkanols (“aliphatic acetogenins”), terpenoid glycosides, various furan ring-containing derivatives, flavonoids, and a coumarin. The highly functionalized alkanols of avocado have exhibited quite diverse biological properties thus far (Yasir et al., 2010[151]). 

Several reports showed that *P. americana *had not only anti-obesity activity, but also positive action on metabolic related alterations. For example, in alloxan-diabetic rats, the administration of a single dose of *P. americana* aqueous extract (100-200 mg/kg) improved the concentration of blood glucose after 12 h of experimentation (24, 22 and 32 % less at 100, 150, and 200 mg/kg, respectively). In addition, same extract doses produced a sustained significant antidiabetic activity during prolonged treatment (12 days) (50, 58 and 60 % less on faster glucose levels, respectively) compared to the control group (Antia et al., 2005[5]).

Similar studies showed that aqueous and methanolic extracts from *P. americana *leaves (10 mg/kg of body weight) for 8 weeks in albino rats with hypercholesterolemia (diet containing 20 % groundnut oil, 0.5 % cholesterol, and 0.25 % cholic acid) improved blood glucose (16 and 11 %, respectively) and lipid profile (total cholesterol: 8 and 5 %; LDL:19 and 20 % respectively); this latter compared with hypercolesterolemic control. Also, plasma HDL concentrations increased by 85 and 68 %, respectively, compared to the hypercholesterolemic controls (Brai et al., 2007[20]). 

Finally, in clinical trials, Ester et al. (2009[42]) reported that the consumption of avocado (30 cm^3^ of avocado-fat) by subjects with dyslipidemia as food for 30 days, decreased triglycerides in serum (10.3 %) and increased HDL (6.3 %) compared with baseline. However, the possible mechanism by which this effect is exerted has not been yet reported, and further studies are suggested.

### Phaseolus vulgaris

Common beans (*Phaseolus vulgaris* L.) are considered an important source of protein in Latin-American countries. For this reason is an important grain legume that comprises 50 % of consumption around the world (Broughton et al., 2003[22]).

Anti-obesity effect has been reported for this grain (Carai et al., 2011[25]). Administration in a multiple cycle of repeated treatments with a standardized *P. vulgaris* dry extracts (50 and 500 mg/kg) in three 5 day treatment periods followed by three 20 day off-treatment periods, resulted in dose-dependent decreases in daily food intake (approx. 17 % at 50 mg/kg dose and 33 % at 500 mg/kg dose) and body weight (approx. 40 % at both doses).

In other recent study, Zhu et al. (2012[157]) reported that consumption of common beans on mice decreased body weight through reduction of plasma leptin concentrations (Zhu et al., 2012[157]). Although differences in final body weights between groups were not statistically significant.

On clinical trials, Celleno et al. (2007[26]) reported that dietary supplement containing 445 mg of *P. vulgaris* extract, after 30 days of treatment, in subjects with a carbohydrate-rich, 2000 to 2200-calorie diet, had significant reductions of body weight (4 %), fat mass (10 %), and waist/hip circumferences (3/1.3 %, respectively) compared with the baseline.

One of the anti-obesity mechanisms reported for *P. vulgaris* relies on the reported α-amylase-inhibiting activity (Celleno et al., 2007[26]). On *in vitro* assays of *P. vulgaris* extracts inhibits activity of α-amylase which could be related with an interfered digestion of complex carbohydrates to simple absorbable sugars, potentially reducing carbohydrate-derived calories (Shamki et al., 2012[125]). Another anti-obesity mechanism reported by Zhu et al. (2012[157]) was the observed regulation of lipid biosynthesis in diet-induced obese C57BL/6J mice treated with cooked dry beans. Others authors, such as Carai et al. (2011[25]) and Spadafranca et al. (2013[132]) concluded that the main anti-obesity activity is the inhibition of appetite improved the obesity conditions.

*P. vulgaris* is an important source of protein, complex carbohydrates, minerals, and dietary fiber. Several bioactives compounds have been reported from common beans such as enzyme inhibitors, lectins, phytates, oligosaccharides, and polyphenols. These compounds may play a key role in human metabolism (Díaz-Batalla et al., 2006[35]). Some phenolic compounds previously identified in *P*. *vulgaris* with biological activity are: quercetin, kaempferol, p-coumaric acid, ferulic acid, p-hydroxybenzoic acid, and vanillic acid (mean contents 10.9, 52.3, 10.1, 9.6, 5.4, and 18.2 µg/g, respectively), raffinose, stachyose, verbascose, and phytic acid (mean contents 8.5, 56.3, 5.5, and 11.5 mg/g, respectively) (Díaz-Batalla et al., 2006[35]).

Besides its anti-obesity effect, it has been reported as a modulation of cardiovascular risk factors. For example, Zhu et al. (2012[157]) reported that short-term feeding of a bean-containing diet (30 and 60 %) reduced plasma total cholesterol (1 and 16 %, respectively) and LDL (21 and 37 %, respectively) levels without affecting HDL or total triglycerides in diet-induced obese C57BL/6J mice. Similarly, Hernández-Saavedra et al. (2013[61]) reported that diet supplemented with 25 % cooked black *P. vulgaris* (N8025) flour in streptozotocin-induced diabetic rats significantly reduced glucose (22.8 %), triglycerides (21.9 %), total cholesterol (29.9 %) and LDL (56.1 %), correlating with a protection of pancreatic ß-cells compared with the diabetic control.

Other research on anti-inflammatory activity of *P. vulgaris* (two cultivars: *Negro 8025* and *Pinto Durango*) showed improved markers of inflammation in lipopolysaccharide (LPS)-induced RAW 264.7 macrophages (Oseguera-Toledo et al., 2011[105]). They also reported that alcalase hydrolysates of common beans var. *Pinto Durango* at 120 min inhibited inflammation, with IC_50_ values of 34.9, 13.9, 5.0, and 3.7 µM, while var. *Negro 8025* needed 43.6, 61.3, 14, and 48.2 µM for the inhibition of cyclooxygenase-2 expression, prostaglandin E2 production, eNOS expression and nitric oxide production, respectively. In addition, the transactivation of NF-kB and nuclear translocation of the NF-kB p65 subunit inhibition through hydrolysates was observed. 

### Capsicum annuum

*Capsicum* species are used mostly as food; furthermore, a medicinal use was also reported, for example: as alternative of treatment for gastric ulcers, rheumatism, alopecia, and diabetes, among others (Tolan et al., 2004[138]). Several studies have reported anti-obesity activity by *Capsicum annuum. *Jeon et al. (2010[69]) reported an anti-adipogenesis activity of methanol extract (doses 50, 100, and 200 μg/mL) from *C. annuum* seeds on adipocyte. They observed a lower lipid accumulation in these cells measured by color changes and compared with the control. Additionally, methanol extracts of *C. annuum* seeds also down-regulated the expression of adipogenic trascriptor factors. Similar effects were observed on animal models. Kim and Park (2014[78]) evaluated green pepper juice for its potential to reduce weight gain and to determine its effects on lipid profiles in C57BL/6L mice fed at high-fat diet. Their results showed that mice fed at 45 % high-fat diet supplemented with green pepper juice (10 mL/kg/day) decreased 16 % their body weight gain compared with the obese controls. 

On clinical trials, capsaicinoids from *Capsicum* were administrated to subjects (age 42 years and BMI 30.4) in dose of 6 mg/day for 12 weeks, causing an abdominal adiposity reduction (21.1 %) than the placebo group (20.2 %), and this effect was positively correlated with the change in body weight.

Among the anti-obesity mechanisms reported for *C. annuum,* the effect on fat oxidation was observed (Snitker et al., 2009[130]); this is due to the stimulation of catecholamine secretion, promoting energy expenditure, and reducing the accumulation of body fat mass (Powell, 2010[113]). On the other hand, Baek et al. (2013[7]) reported inhibitory effects of *C. annuum *water extracts on lipoprotein lipase activity in 3T3-L1 cells, decreasing the mRNA expression level to 50.9 % compared with the control group.

The chemical composition of *C*. *annuum *includes compounds such as capsaicin and several related capsaicinoids, cinnamic derivatives, trigonelline, C4-substituted pyridine, amino acids, small organic acids, and fatty acids (Ritota et al., 2010[120]). Additionally, *C*. *annum* is considered a good source of vitamins C and E, provitamin A, carotenoids and various phenolics and flavonoids (Materska and Perucka, 2005[89]).

Besides its anti-obesity activity, C. *annuum *significantly decreased cardiovascular risk factors such as serum triglycerides (31 %), total cholesterol (12 %), and low density lipoproteins (36 %) in mice fed with a high-fat diet and supplemented with green pepper juice (10 mL/kg/day). Magied et al. (2014[87]) reported that male albino rats containing 20 % high fat diet rendered diabetic with alloxan injection for 4 weeks and treated with 0.015 % capsaicin, decreased their blood serum glucose (50 %), serum cholesterol (48 %), and serum triglycerides (69 %) compared with the diabetic control.

### Rosmarinus officinalis

Rosemary (*Rosmarinus officinalis* L.) from the family Labiatae is an evergreen perennial shrub cultivated in many parts of the world and widely used as a spice, food supplement and for cosmetic applications (Aguilar et al., 2008[1]; Harach et al., 2010[56]). Traditionally, *R. officinalis* has been used in renal colic as an antispasmodic, to relieve dysmenorrheal symptoms (Takaki et al., 2008[134]) and respiratory disorders (Fabio et al., 2007[43]).

Several studies report the effect of *R. officinalis* to promote weight loss. Harach et al. (2010[56]) experimented with *R. officinalis* leaf extracts administered for 50 days at doses to 200 mg/kg of body weight in mice fed a high-fat diet, which induced a significant reduction of weight and fat mass gain (64 and 57 %, respectively). Similarly, Ibarra et al. (2011[68]) reported that rosemary leaf extract standardized to 20 % carnosic acid was administered (500 mg/kg body weight/day) in C57BL/6J mice fed with high-fat-diet, reducing body and epididymal fat (69 and 79 %, respectively) compared with an obese control. 

The main anti-obesity activity reported for *R. officinalis *is that it increases fecal fat excretion without decreasing food intake (Harach et al., 2010[56]; Ibarra et al., 2011[68]). In concordance, Bustanji et al. (2010[23]) reported an *in vitro* inhibitory effect of *R. officinalis* on hormone sensitive lipase and pancreatic lipase. The highest effect was for pancreatic lipase (IC_50_: 13.8 µg/mL) than hormone sensitive lipase (IC_50_: 95.2 µg/mL), suggesting that extracts of *R. officinalis *had more affinity for the first one.

Another anti-obesity mechanism for *R. officinalis* is by its antiadipogenic activity. Gaya et al. (2013[49]) reported that carnosic acid (5 μg/ml), the main bioactive compound of *R. officinalis* extract, inhibits 3T3-L1 preadipocytes differentiation. This inhibition was accompanied by a blockade of mitotic clonal expansion.

Besides carnosic acid, other compounds present in *R. officinalis* are monoterpenes, diterpenes and phenolics. The main polyphenols identified are caffeic acid derivates such as rosmarinic acid (ester of caffeic acid and α-hydroxydihydrocaffeic acid) and α-hydroxydihydrocaffeic acid, chlorogenic acid, and their hydrolyzed metabolites (El Deeb, 1993[40]; Herrero et al., 2010[62]; Rababah et al., 2004[116]; Wang et al., 2004[143]). 

In addition to its anti-obese activity, rosemary leaf extracts standardized to 20 % carnosic acid in obese mice, also experienced 72 % less in plasma glucose levels and 68 % less in total cholesterol compared with the control (Ibarra et al., 2011[68]). 

On clinical trials, Labban et al. (2014[79]) reported that 10 g/day of rosemary leaves powder for 4 weeks in men and women aged 20 to 57 years, significantly reduced fasting serum glucose (18 %), total cholesterol (34 %), LDL concentration (34 %), triglycerides (29 %), and malondialdehyde (36 %) compared with the baseline. 

These results suggest that rosemary is a good natural alternative for obesity and its metabolic alterations. 

### Ilex paraguariensis

Yerba mate (*Ilex paraguariensis*) is a native plant from the subtropical regions and one of the most consumed in South America. Its cultivation is done in Brazil, Argentina, Uruguay and Paraguay. Its leaves are used to prepare different beverages (Lima et al., 2014[83]). Traditionally, they are used as mild central nervous system stimulant, diuretic, and in weight reducing preparations (Pang et al., 2008[107]).

Recently, *I. paraguariensis *has been studied due to its potential beneficial effects in the treatment of obesity. Kang et al. (2012[72]) reported that treatment of *I*. *paraguariensis* dried aqueous extract (2 g/kg of body weight) in C57BL/6J mice fed with high-fat diet affected food intake (-20 %), resulting in higher energy expenditure and losing body weight (23 %). Similarly, Lima et al. (2014[83]) reported that administration of an instant *I*. *paraguariensis* solution (1 g/kg body weight) once a day during 30 days by intragastric gavage in obese rats primed by early weaning, decreased their body weight (11 %) and food intake (25 %) compared with the control. 

The effect on food intake is considered the main anti-obesity mechanism of *I*. *paraguariensis.* This improved the content of neuropeptide Y and may contribute to the correction of hyperphagia, independently of the leptin action (Seo et al., 2008[124]). 

Focusing on the chemical composition, *I. paraguariensis* included flavonoids (quercertin, rutin, and kaempferol) (Pomilio et al., 2002[111]), caffeoyl derivatives (caffeic acid, chlorogenic acid, 3, 4-dicaffeoylquinic acid, 3, 5-dicaffeoylquinic acid, and 4, 5-dicaffeoylquinic acid), methylxanthines (caffeine, theophylline, and theobromine), tannins, and numerous triterpenic saponins that are derived from ursolic acid and are known as metesaponins (Andersen and Fogh, 2001[4]; Pomilio et al., 2002[111]). The polyphenol content in *I. paraguariensis* is higher than in green tea (Bixby et al., 2005[17]).

*In vivo* research showed that *I. paraguariensis* has positive effects on metabolic alterations, consequence of obesity, including reductions in serum cholesterol, serum triglycerides, and glucose concentrations. Kang et al. (2012[72]) reported that treatment of obese mice with *I*. *paraguariensis* dried aqueous extracts, decreased the faster glucose (27 %) and total cholesterol (10 %) levels; whereas, Lima et al. (2014[83]) reported a reduction in serum triglycerides (31 %), faster glucose (12 %), and serum insulin (14 %) in obese rats treated with instant yerba mate solution.

On the other hand, Lima et al. (2014[84]) reported that administration of *I*. *pararguariensis *aqueous solution (1 g/kg body weight/day, gavage) for 30 days in obese rats primed by early weaning improved inflammatory profile (decreased TNF-α < 50 %, and increased IL-10 < 50 %) compared with their counterpart control.

### Citrus paradisi

Grapefruit (*Citrus paradisi*) was first discovered in the forests of Caribbean island, Barbados. It is now one of the widely cultivated fruits in the United States, particularly in Florida, California, and the other semi-tropical Southern states. The fruit is a natural hybridization of pomelo and orange. The plant is a subtropical citrus tree and botanically belongs to the large Rutaceae family of citrus fruits from the genus Citrus (Bailey, 2004[8]). 

Although, grapefruits are traditionally used for losing weight, there are only a few studies related to this activity. Gamboa-Gómez et al. (2014[47]) reported that treatment with *C. paradisi* infusion (*ad lib.*) on obese rats fed with a high-saturated-fat-diet exhibited a reduction in adipocyte size and volume. These results were in agreement with triglycerides levels on adipose tissue, which were reduced in 16 % compared with obese controls.

On the other hand, in a clinical trial, Fujioka et al. (2006[45]) reported an anti-obesity effect of grapefruit fresh, grapefruit juice and grapefruit capsules in obese patient with diagnosed metabolic syndrome. Ninety-one humans were included in 4 groups: placebo (placebo capsules and 207 mL of apple juice), group 2: (grapefruit capsules with 207 mL of apple juice), group 3 (237 mL of grapefruit juice with placebo capsule), and group 4 (half of a fresh grapefruit with a placebo capsule) three times a day before each meal. After twelve weeks of treatment group 4 had the highest effect decreasing 1.6 kg, followed by group 3, and group 2 with 1.5 and 1.1 kg respectively. Similarly, Dow et al. (2012[37]) reported that one half of a fresh Rio-Red grapefruit with each meal (3Xdaily) for 6 weeks was associated with modest weight loss (3 %) on overweight/obese subjects compared with control for 6 weeks.

For the possible anti-obesity mechanism of *C. paradisi* Gamboa-Gómez et al. (2014[47]) reported that *C. paradisi* infusion regulates the lipid metabolism, inducing relative expression of carnitine palmitoyl-transferase 1a (CPT1a) in obese rats compared with the control. CPT1a is responsible for the transportation of long chain fatty acids into the mitochondria through binding to carnitine, and then moving into the route of β-oxidation. 

Active compounds reported for *C. paradisi* including flavonoids (naringin and hesperidin, lycopene, among others) and furanocoumarins (bergamottin and 6',7'-dihydroxybergamottin) (De Castro et al., 2006[32]; Gattuso et al., 2007[48]), β-carotene, and *d*-limonene were also reported (Vanamala et al., 2005[140]).

Not only anti-obesity activity was reported for *C. paradisi*, but also an improved effect on cardiovascular and diabetes risk factors. Gamboa-Gómez et al. (2014[47]) reported that treatment with *C. paradisi* infusion improved faster glucose concentration (28 %), insulin (37 %), and total triglycerides (26 %) in obese rats. The treatment with *C. paradisi* infusions improved, as well as hepatic oxidative stress markers such as thiobarbituric acid reactive substances (82 %) and carbonyl content of proteins (32 %) compared with the obese control. 

Same behavior was observed in clinical trials. Dow et al. (2012[37]) reported improvements in circulating lipids in overweight adults that consumed grapefruit, with total cholesterol and LDL levels significantly decreasing, compared with baseline values.

### Citrus limon

Lemon plant (*Citrus limon* L.) belongs to the Rutaceae family, and is the third most important Citrus species after orange and mandarin. Crude extracts of different parts of lemon (leaves, stem, root and flower) have anticancer activities and antibacterial potential against clinically significant bacterial strains (Kawaii et al., 2000[75]).

On obesity research, Fukuchi et al. (2008[46]) reported that dietary lemon polyphenols extracted from lemon peel (0.5 % w/w) on high-fat diet-induced obesity in C57BL/6J mice for 12 weeks suppressed body weight gain (44 %) and body fat accumulation (36 %).

One anti-obesity mechanism reported for lemon is by up-regulation of peroxisomal β-oxidation through the increase mRNA level of acyl-CoA oxidase in the liver and white adipose tissues, which was likely mediated via up-regulation of the mRNA levels of peroxisome proliferator activated receptor-α (PPARα) (Fukuchi et al., 2008[46]).

*Citrus limon* contains many important phytochemicals, including phenolic compounds (mainly flavonoids) and other nutrients and non-nutrients (vitamins, minerals, dietary fiber, essential oils and carotenoids) (González-Molina et al., 2008[50]). These compounds are not equally distributed in the lemon fruit. Hesperidin and eriocitrin occur mainly in lemon juice; two isomers of hesperidin, neohesperidin and homoeriodictyol 7-O-rutinoside have also been identified in lemon juices (González-Molina et al., 2008[50]; Wang et al., 2008[144]). Lemon seeds are rich in eriocitrin, hesperidin and in less amounts in naringin; whereas, the peel is rich in neoeriocitrin, neohesperidin and naringin and has minor amounts of narirutin (Baldi et al., 1995[9]).

Additionally, it has been reported that *C. limon* has effects on metabolic alterations caused by obesity. Fukuchi et al. (2008[46]), reported that supplementation with lemon polyphenols on high-fat diet-induced obesity in mice significantly improved hyperlipidemia (serum triglycerides -18 % , total cholesterol -26 %, and serum free fatty acids -5 %), hyperglycemia (insulin -65 % and faster glucose -26 %), and insulin resistance (-75 %) than obese controls.

Similarly, Naim et al. (2012[102]) reported that hexane extracts from lemon peels showed antidiabetic activity in alloxan-induced diabetic rats, detecting reduced blood glucose level of 44.57, 75.96, 95.43, and 98.08 % in 24, 48, 72, and 96 h, respectively, compared with counterpart controls.

Moreover, there are no reports on the anti-obesity effect or its associated alterations of *C. limon* in clinical studies.

### Punica granatum

Pomegranate (*Punica granatum* L.) is a small tree, belonging to the Punicaceae family. Pomegranate, especially its fruit, possesses a vast ethnomedical history and represents a phytochemical reservoir of heuristic medicinal value (Lansky and Newman, 2007[80]). Biological studies about pomegranate juice have shown antidiabetic effect (Katz et al., 2007[74]). This activity is credited to the pomegranate's high antioxidant capacity, which can be attributed to its high content of polyphenols.

On obesity research, Lei et al. (2007[81]) reported that treatment with pomegranate leaf extracts (400 and 800 mg/kg/day) for 5 weeks in obese mice feed with a high-fat diet significantly decreased body weight (12 and 20 %, respectively), energy intake (14 and 30 %, respectively), and various adipose pad weight (14 and 42 %, respectively) than obese controls.

Another study reported an anti-obesity effect of pomegranate seed oil in male C57BL/6J mice fed on a high-fat diet (1 g/100 g) for 12 weeks. Treatment decreased body weight (-10 %) and fat mass (50 %), whereas no differences in lean mass were observed. The latter compared with an untreated obese (Vroegrijk et al., 2011[142]).

On the other hand, Xu et al. (2009[150]) reported that male Zucker diabetic fatty rats treated with pomegranate flower extract (500 mg/kg) per 6 weeks, ameliorates diabetes and obesity-associated fatty liver, reducing ratio of liver weight to tibia length, hepatic triglyceride contents and lipid droplets. 

The anti-obesity mechanism reported for pomegranate flower is at least in part, by activating hepatic expression of genes responsible for fatty acid oxidation. Other anti-obesity mechanisms reported for *P. granatum* are inhibition of the pancreatic lipase activity, suppressing energy intake by appetite suppressant (Lei et al., 2007[81]). 

Pomegranate extracts (juice, seed oil, and flower extracts) are rich in many compounds such as proanthocyanidin flavonoids, and ellagitannins; additionally, several polysaccharides, and many minerals including potassium, nitrogen, calcium, magnesium, phosphorus, and sodium (Viuda-Martos et al., 2010[141]). The pomegranate seeds contain high concentration of conjugated fatty acids such as linoleic and linolenic acids and other lipids: punicic acid, stearic acid, palmitic acid, and phytosterols. Minor amounts of conjugated linolenic acid isomers including eleostearic acid and catalpic acid, and phytosterols are also found (Vroegrijk et al., 2011[142]). It has been identified tannins, anthocyanins, vitamin C, vitamin E, coenzyme Q10, and lipoic acid in pomegranate juice. Compounds such as anthocyanins, are responsible for the fruit color, being the most important phenolic group present in the arils or juice (Viuda-Martos et al., 2010[141]).

It has been reported that pomegranate extracts possess not only anti-obesity effect, but also action on the metabolic alterations associated to this disease. For example, Eidi (2014[39]) reported that hydro-ethanolic extract of *P. granatum* flowers at 200 and 300 mg/kg doses in streptozotocin-induced diabetic rats administered intraperitonally for 18 days, significantly reduced the serum glucose (37 % at both doses), cholesterol (14 % at both doses), triglycerides (33 and 16 %, respectively), LDL (50 % at both doses), urea (approx. 58 % at both doses), uric acid (40 % at both doses), creatinine (30 % at both doses), alanine amino transferase (approx. 27 % at both doses) and aspartate amino transferase enzymes levels (approx. 27 % at both doses), while it increased serum HDL (14 %) level in comparison to control diabetic rats.

On clinical trials, Mirmiran et al. (2010[92]) reported that consumption of pomegranate seed oil (400 mg) twice daily, for 4 weeks by hyperlipidaemic subjects, diagnosed according to National Cholesterol Education Program definition, decreased the total concentration of serum triglycerides (20 %) compared with baseline values.

Moreover, more detailed molecular/cellular studies in humans are needed to relate the anti-obesity potential of the fruit as common consumed by population.

### Aloe vera

*Aloe vera* belongs to the family Liliaceae, and it is widely used in the manufacturing of food and drink products, pharmaceuticals, and cosmetics (Reynolds and Dweck, 1999[118]). *Aloe* species have been used around the world because of their antitumor, anti-infection, anti-inflammatory, antioxidant, and laxative effects (Prabjone et al., 2006[114]; Yu et al., 2009[154]).

Recent research studies have reported that *Aloe vera* has anti-obesity effect. Misawa et al. (2012[93]) studied the effect of *A. vera* gel powder administration in Sprague-Dawley rats with diet-induced obesity at two doses (20 and 200 mg/kg/day) for 90 days. They observed a modest decrease in body weight, but a significantly reduced subcutaneous (approx. 41 % at both doses) and visceral fat weight (16 and 30 %, respectively) compared with the obese control.

Misawa et al. (2012[94]) reported that oral administration of lophenol and cycloartanol, two kinds of phytosterols isolated from *A. vera* (25 μg/kg/day) once a day for 44 consecutive days in Zucker diabetic fatty rats significantly reduced visceral fat weights (26 %) than obese control.

In clinical trials, Choi et al. (2010[29]) reported that treatment of pre-diabetes or early diabetes mellitus volunteers, with two capsules (147 mg/capsule) of *A. vera* gel complex after breakfast and two more after dinner for 8 weeks reduced body weight (2.5 %) and body mass fat (6.2 %) than the placebo group. Additionally, volunteers improved concentrations on fast glucose (2 %), insulin (8 %), and insulin resistance (11 %) compared with baseline values.

One proposed anti-obesity mechanism for *A. vera* is in part, by stimulation of energy expenditure (Misawa et al., 2012[93]). Other anti-obesity mechanism reported for *A. vera* is the regulation of expression levels of hepatic genes encoding to lipogenic enzymes (ACC, FAS), and transcriptor factor SREBP-1, which decreased significantly by the administration of aloe sterols; and to the increased of hepatic β-oxidation enzymes ACO, CPT1, PPARα (Misawa et al., 2012[94]).

The chemical composition of *A. vera *includes components such as phytosterols, namely, lophenol, 24-methyllophenol, 24-ethyllophenol, cycloartanol, and 24-methylenecycloartanol, anthraquinones, carbohydrates, chromones, enzymes, inorganic compounds, lipids, tannins, amino acids, proteins, saccharides, vitamins, pectins, hemicelluloses, glucomannan, acemannan, and mannose derivatives (Misawa et al., 2012[93]).

Several studies have shown beneficial effects of *A. vera* not only on obesity, but also on the associated metabolic disorders. For example, Mohamed (2011[95]) reported that oral administration of *A. vera* gel extract (0.5 mL/day for 6 weeks) in alloxan induced diabetic rats resulted in a significant reduction of serum glucose (52 %), total cholesterol (31 %), and triglycerides (20 %). The latter compared with an untreated diabetic control. On the other hand, the treatment with *A. vera* gel extract improved the oxidative stress condition evidenced by a significant decrease in serum malondialdehyde levels, a significant increase in serum nitric oxide, and antioxidant activity in treated diabetic group as compared with diabetic control group.

Similarly, Huseini et al. (2012[67]) reported that hyperlipidemic (hypercholesterolemic and/or hypertriglyceridemic) type 2 diabetic patients aged 40 to 60 not using other anti-hyperlipidemic agents, have tried *A. vera* gel capsules (300 mg/12 h/ for 2 months), lowering significantly their total cholesterol, LDL concentrations, blood glucose, and glycosylated hemoglobin (HbA1c) levels and no significant effects in liver and kidney functions were noted compared with the control.

### Taraxacum officinale

Dandelion (*Taraxacum officinale*), is a member of the Asteraceae/Compositae family. It has been considered as an herbal medicine due to its antidiabetic, choleretic, and diuretic properties (Schütz et al., 2006[123]). Several studies reported that treatment with *T. officinale* could improve health, decreasing inflammation and tumors (Kim et al., 2007[76]; Sigstedt et al., 2008[127]). 

There is little information on the anti-obesity effect of dandelion; however, just recently and despite not being the main objective, Davaatseren et al. (2013[31]) found that *T. officinale* leaf extract (2 and 5 g/kg) on high-fat-diet-induced C57BL/6J mice reduced the body weight (12 and 7 %, respectively) compared with the obese control. 

The main anti-obesity mechanism reported for *T. officinale *was the inhibition *in vitro* and *in vivo* of pancreatic lipase (Zhang et al., 2008[156]); however, further studies are required leaving an open field to explore. 

The chemical characterization of *T. officinale* includes luteolin-7-glucoside and apigenin-7-glucoside from leaf tissue (Wolbis and Krolikowska, 1985[146]; Wolbis et al., 1993[147]). Also, free quercetin and luteolin, luteolin-7 and 4'-glucosides, luteolin-7-rutinoside, quercetin-7-glucoside and isorhamnetin-3-glucoside and 3,7-diglucoside are present in combined leaf and flower extracts. Additionally, caffeic acid was reported (Wolbis et al., 1993[147]), chlorogenic acids, and monocaffeyltartaric acid from the leaf, roots and flowers, and p-hydroxyphenylacetic acid from both leaf and root tissues (Williams et al., 1996[145]). 

Although, little information on body and fat weight effects is available, several studies report the effect of *T. officinale *on alterations associated with obesity. For example, Choi et al. (2010[29]) reported hypolipidemic effects of dandelion leaf (1 % w/w) in rabbits fed with a high-cholesterol diet. They reported an improvement on lipid profile (-36 % on triglycerides, -11 % on LDL values, and 29 % higher of HDL concentration) of treated rabbits compared with the control. Additionally, they reported an improvement on the thickness of aorta (58 % less than control).

More recently, Davaatseren et al. (2013[31]) reported that *T. officinale* leaf extract (2 and 5 g/kg) on high-fat-diet-induced C57BL/6J mice improved faster glucose (approx. 15 %), insulin (approx. 43 %), and insulin resistance (-49 %) than control.

### Arachis hypogaea

Peanuts (*Arachis hypogaea* L.) belong to the Fabaceae family. Its cultivation is relevant worldwide, being the fourth largest edible oilseed crop in the world (Shilman et al., 2011[126]). The primary use of peanuts is as food either as shelled nuts or as a source for seed oil. Peanut shells are considered as a non-value contaminant residue, with thousands of tons produced annually by the peanut industry (Sobolev and Cole, 2004[131]).

It has been reported that frequent nut consumption, including peanuts, was associated with a significantly lower risk of coronary heart disease (Hu et al., 1998[64]).

On obesity research, Moreno et al. (2006[98]) reported that ethanolic peanut shell extracts (1 % of extracts w/w, 85 days) reduced body weight (10 %) in high-fat diet male Wistar rats compared with the obese control.

In a more recent study, Kang et al. (2014[71]) studied the effect of high fat diets with low and high peanut sprout extract diets (20 % fat and 0.025 or 0.05 % peanut sprout extract) on Sprague-Dawley rats for 9 weeks, resulting in a decreasing body weight gain compared with the obese group. No statistical differences were reported for food consumption between experimental groups. In addition, epididymal fat weight in high peanut sprout extract group (3.61 g), or low peanut sprout extract group (3.80 g), was significantly lower than the control obese group (4.39 g). In the same experiment the authors found less total triglycerides (9.5 and 12 % respectively) and total cholesterol (12 and 13 %, respectively) than in the control group.

Moreno et al. (2006[98]) attributed the anti-obesity effect of *A. hypogaea* to its inhibition of pancreatic lipase and other gastro-intestinal lipases; they observed an increased fecal lipid content, decreasing the digestibility of dietary fat. Whereas Kang et al. (2014[71]) attributed the anti-obesity activity of *A. hypogaea* to the effect on expression of PPARγ. This is an adipogenic transcription factor, and the target gene adiponectin. They suggest that up-regulation of PPARγ and adiponectin consequence of the consumption of peanut sprout extract in rats could be associated with the reported lower epidermal fat and total weight gain. Peanut shells (hulls, seed coats) contain several bioactive molecules, such as luteolin, certain fatty acids, caffeic, ferulic and benzoic acids, all of which are able to inhibit lipases (Birari and Bhutani, 2007[16]). Grosso and Guzman (1995[52]) reported the following lipids on peanuts: Palmitic, stearic, oleic, linoleic, arachidic, eicosenoic, behenic, among others. The sterol composition in peanut is β-sitosterol, campesterol, stigmasterol and Δ^5 ^-avenasterol (Grosso et al., 2000[53]). Besides the anti-obesity effect of *A. hypogaea*, it has been reported with hypoglycemic, hypolipidemic, and hypertensive activities. Bilbis et al. (2002[15]) reported that aqueous extract of *A. hypogaea* seeds (with free access to the extract as the only drinking water for 21 days) in normal and alloxan-induced diabetic rats decreased the fasting blood glucose level (189.0 to 107.55 mg/dL), and improved their lipid profile (14 % on total triglycerides, 11 % on total cholesterol, and 17 % in LDL concentration) compared with diabetic control. 

Similarly, Kang et al. (2014[71]) found an improvement in lipid profile in rats fed with high fat diet using low and high peanut sprout extract diets (20 % fat plus 0.025 or 0.05 % peanut sprout extract).

On the other hand, Quist et al. (2009[115]) reported that raw and roasted peanut flour, proteolytic digested, have an inhibitory effect on the activity of angiotensin converting enzyme (ACE) *in vitro*, exhibiting for alcalase digestion of raw peanut IC_50_ values of 8.7-122 mg/mL, and from roasted flour 12-235 mg/mL. More *in vivo* research is needed to expand on this effect.

## Conclusions

Obesity is a significant and increasing public health problem worldwide. Even though, there are several treatments, such as surgery and drugs, there seems to be no efficient treatment without potential side effects; thus, considering a lifestyle modification as the best option. In addition to a lifestyle modification, natural alternatives may provide increased health expectancy. As we have summarized, several plants possess anti-obesity potential and have been poorly studied, while others are not even promoted. 

More anti-obesity data is needed, but in order to accomplish this, more research in this area with well-designed clinical trials focused on both safety and efficacy with some of these plants materials is required.

## Acknowledgements

The authors gratefully acknowledge Bogar M. Vallejo and tradu-c services for language revision.
